# Editorial: Imaging brain molecular connectivity in health and disease

**DOI:** 10.3389/fnhum.2023.1298382

**Published:** 2023-10-10

**Authors:** Arianna Sala, Kristina Herfert, Gabriel Gonzalez-Escamilla, Silvia Paola Caminiti, Daniele Bertoglio

**Affiliations:** ^1^Coma Science Group, GIGA-Consciousness, University of Liège, Liège, Belgium; ^2^Centre du Cerveau^2^, University Hospital of Liège, Liège, Belgium; ^3^Department of Preclinical Imaging and Radiopharmacy, Werner Siemens Imaging Center, Eberhard Karls University Tübingen, Tübingen, Germany; ^4^Movement Disorders and Neurostimulation, Department of Neurology, University Medical Hospital of the Johannes Gutenberg University Mainz, Mainz, Germany; ^5^Department of Brain and Behavioral Sciences, University of Pavia, Pavia, Italy; ^6^Bio-Imaging Lab, University of Antwerp, Antwerp, Belgium; ^7^μNEURO Research Centre of Excellence, University of Antwerp, Antwerp, Belgium

**Keywords:** PET, SPECT, MRI, system neuroscience, metabolic connectivity, resting-state networks, structural connectivity

Over recent decades, advances in the field of neuroimaging have pushed forward our understanding of the central nervous system in health and disease. Contemporary neuroimaging techniques such as functional magnetic resonance imaging (fMRI) and electroencephalography (EEG) have made essential contributions to study the brain from a functional perspective, with growing emphasis on brain networks and connectivity. Brain molecular imaging, including positron emission tomography (PET) imaging and single-photon emission computerized tomography (SPECT), is a unique molecular tool to obtain direct quantitative information on biological processes such as neural activity, neuroreceptor distributions, and proteinopathies, covering a wide range of (patho)physiological processes occurring during development, aging, and neurological and neuropsychiatric disorders. Despite the development of methods and tools to analyze molecular neuroimaging data with multivariate connectivity methods, many molecular imaging studies still predominately use univariate approaches. The application of either new methods of analysis or novel applications of new imaging technologies using statistical multivariate approaches to extract brain connectivity could enrich connectivity estimates, thus providing valuable insights into brain metabolic and molecular features. This Research Topic aimed at gathering research focusing on the investigation of brain connectomics from a molecular imaging perspective in health and disease.

Resting-state network (RSN) connectivity is widely used for understanding the brain's functional organization. In this line, Fang et al. investigated associations between RSN connectivity and synaptic density assessed using the synaptic vesicle glycoprotein 2A radioligand [^11^C]UCB-J PET. RSN connectivity was investigated based on the fractional amplitude of low-frequency fluctuations (fALFF), a common measure of resting-state fMRI signal magnitude. This work suggests that RSN functional connectivity may be linked to synaptic architecture through multiple local and circuit-based associations.

To relate to the brain's structural organization, Jensen et al. investigated associations between longitudinal variations of gray matter networks (inter-subject covariance of brain volume changes) and epigenetic expression (DNA methylation) in the adolescent brain. This work reports longitudinal gray matter changes in late childhood and early adolescence in seven brain networks and links them to changes in the expression of three genes related to myelination, excitatory and inhibitory receptors, and connectivity. Moreover, the authors report trending associations between GM network volume changes cognitive changes in crystallized and fluid cognition, as well as episodic memory and attention/inhibitory control. The study highlights the utility of combining structural and molecular data to detail our understanding of the underpinnings of connectivity dynamics in the adolescent brain.

The advent of novel imaging hardware such as hybrid PET-MR imaging now provides the opportunity for the generation of multidimensional data to parallel map brain network connectivity measures at multiple spatial and temporal scales. Leveraging the power of PET-MR systems, Devrome et al. report a combined evaluation of brain network connectivity using structural (diffusion-weighted MRI), functional (resting-state fMRI), and metabolic ([^18^F]FDG PET) data. The different networks were then combined into a multiplex core. By comparing young and elderly healthy volunteers, this work highlights the potential of multiplex networks as an integrative approach to capture the relevant information in multimodal neuroimaging data.

Beyond its value in understanding brain variations during development or brain states, a number of contributions to this Research Topic highlighted the value of imaging molecular connectivity in the context of pathological conditions. For instance, Huang et al. conducted a multiparameter quantitative evaluation of the brain metabolic network changes using [^18^F]FDG PET in patients with anti-N-methyl-D-aspartate receptor (NMDAR) encephalitis using a graph theory analysis method to detect specific alterations in connectivity. Their findings indicated that certain characteristics and connections of the metabolic network reflected the clinical status, including a reduced connectivity between occipital and temporal lobes, increased connectivity in the frontal lobe, cingulate gyrus, and caudate nucleus, which could possibly be a compensatory mechanism, and observed distinctions that were lateralized group differences. These insights suggest that the identification of brain metabolic abnormalities can help to improve our understanding of ongoing neuropathological activity in specific patient populations.

In a further exemplary application, in the context of Parkinson's disease (PD), Zang et al. focused on the primary sensorimotor area (PSMA) to explore the abnormal functional connectome and the association to the metabolic network in relation to disease severity. Utilizing a hybrid PET/MR scanner to investigate the disruption of PSMA functional connectome (as from resting state fMRI) and its association with glucose metabolism (as from [^18^F]FDG PET) in 9 RSN of PD patients. By calculating the degree of centrality, the authors identified disease severity-dependent PSMA functional connectome abnormalities in PD patients, mostly involving the visual, attention, somatomotor, limbic, frontoparietal, and default mode networks, which uncoupled with the metabolic network. The functional and metabolic network uncoupling in PD patients, likely driven by decreased functional connectivity but increased FDG uptake, highlights the complementary nature of fMRI and PET measures with possibly differential underlying mechanisms.

The study by Yuan et al. aimed to investigate the relationship between glucose metabolism and functional brain activity in patients with mesial temporal lobe epilepsy (MTLE), a drug-resistant seizure from the hippocampus and related areas, often due to hippocampal sclerosis. Emerging research highlights MTLE as a network-based condition impacting networks such as the default mode network (DMN). Here, the authors used a hybrid PET/MR scanner to explore metabolic-fALFF coupling alterations and to evaluate whether the associations could predict surgical outcomes in these patients. The ipsilateral hippocampus showed decreased metabolic covariance network connectivity, while the contralateral hippocampus exhibited increased connectivity. Patients with different surgical outcomes also had varying metabolic-fALFF couplings within the epileptogenic network. The study discussed potential reasons for the observed coupling changes, including the impact of seizures on neuronal damage and oxidative stress. It also noted that alterations in functional connectivity within the DMN and hippocampal connectivity were linked to seizure generation and MTLE-related impairments. Finally, the coupling between metabolic and functional connectivity in the DMN regions, thalamus and hippocampus was shown as the best predictor of surgery outcomes. These findings suggested that assessing neuroenergetic coupling using PET/MR imaging could enhance our understanding of MTLE pathogenesis and offer a new metric for assessing patients who may better benefit from surgery.

Using an animal model, Puy et al. investigated the effects of intracerebral hemorrhage on brain structure (using ultra-high field MRI) and metabolic network organization. The authors assessed the effect of localized hemorrhage (as induced consistently at the level of the right striatum) on metabolism and metabolic connectivity as well as atrophy and gray-matter covariance at 3 months post-insult, in a rat sample. The authors found severe atrophy and hypometabolism at and beyond the site of insult, accompanied by a decreased in metabolic and gray-matter covariance predominantly in the limbic system connected to the affected site but distant to the initial insult site. The authors also found increased metabolic and gray-matter covariance in the contralateral hemisphere. In summary, the authors suggested that widespread maladaptive and compensation processes coexist in the rat brain following intra-cerebral hemorrhage and interfere with memory domains.

Cerebral perfusion represents an important metric related to brain activation states. Despite the low temporal resolution of SPECT imaging, Doyen et al. showed the potential of dynamic brain perfusion SPECT imaging to extract cerebral resting state networks. In this case report of an 80-year-old man, functional SPECT connectivity (fSPECT) was assessed through a seed correlation analysis, and five well-known resting-state networks were identified: the executive, the default mode, the sensory-motor, the salience, and the visual networks. This case report supports the feasibility of fSPECT imaging in identifying well-known resting-state networks.

In summary, this Research Topic contributes to raising awareness of the promising opportunities that the investigation of molecular brain connectomics can bring to our understanding of the brain in health and disease. Several contributions to this Research Topic highlighted the value of applying an integrative approach combining brain molecular imaging with different MRI-based methods and genetics, as well as possibly electrophysiological tools. Such multimodal approaches are extremely powerful in studying the brain at different temporal and spatial scales, providing valuable insights into brain function and architecture in development, aging, and disease.

## Author contributions

AS: Writing—original draft, Writing—review and editing. KH: Writing—original draft, Writing—review and editing. GG-E: Writing—original draft, Writing—review and editing. SC: Writing—original draft, Writing—review and editing. DB: Writing—original draft, Writing—review and editing.

